# Abcg2 Overexpression Represents a Novel Mechanism for Acquired Resistance to the Multi-Kinase Inhibitor Danusertib in BCR-ABL-Positive Cells In Vitro

**DOI:** 10.1371/journal.pone.0019164

**Published:** 2011-04-26

**Authors:** Stefan Balabanov, Artur Gontarewicz, Gunhild Keller, Laura Raddrizzani, Melanie Braig, Roberta Bosotti, Jürgen Moll, Edgar Jost, Christine Barett, Imke Rohe, Carsten Bokemeyer, Tessa L. Holyoake, Tim H. Brümmendorf

**Affiliations:** 1 Klinik für Onkologie, Hämatologie und Knochenmarktransplantation mit Sektion Pneumologie, Universitäres Cancer Center Hamburg (UCCH), Universitäts-Klinikum Hamburg-Eppendorf, Hamburg, Germany; 2 Nerviano Medical Sciences - Oncology, Nerviano, Milan, Italy; 3 Klinik für Hämatologie und Onkologie, Medizinische Klinik IV, Universitätsklinikum Aachen, Rheinisch Westfälische Technische Hochschule (RWTH) Aachen, Aachen, Germany; 4 Section of Experimental Haematology and Haemopoietic Stem Cells, University of Glasgow, Glasgow, United Kingdom; Cleveland Clinic, United States of America

## Abstract

The success of Imatinib (IM) therapy in chronic myeloid leukemia (CML) is compromised by the development of IM resistance and by a limited IM effect on hematopoietic stem cells. Danusertib (formerly PHA-739358) is a potent pan-aurora and ABL kinase inhibitor with activity against known *BCR-ABL* mutations, including T315I. Here, the individual contribution of both signaling pathways to the therapeutic effect of Danusertib as well as mechanisms underlying the development of resistance and, as a consequence, strategies to overcome resistance to Danusertib were investigated. Starting at low concentrations, a dose-dependent inhibition of BCR-ABL activity was observed, whereas inhibition of aurora kinase activity required higher concentrations, pointing to a therapeutic window between the two effects. Interestingly, the emergence of resistant clones during Danusertib exposure *in vitro* occurred considerably less frequently than with comparable concentrations of IM. In addition, Danusertib-resistant clones had no mutations in *BCR-ABL* or aurora kinase domains and remained IM-sensitive. Overexpression of Abcg2 efflux transporter was identified and functionally validated as the predominant mechanism of acquired Danusertib resistance *in vitro*. Finally, the combined treatment with IM and Danusertib significantly reduced the emergence of drug resistance *in vitro*, raising hope that this drug combination may also achieve more durable disease control *in vivo*.

## Introduction

Inhibition of BCR-ABL tyrosine kinase by Imatinib (IM, formerly STI571, Gleevec®) set new standards in the treatment of chronic myeloid leukemia (CML). Indeed, durable hematological and cytogenetic response can be achieved in the majority of patients [1], [2], [3]. However, primary or acquired resistance to IM remains a major therapeutic challenge in the course of CML treatment, in particular in patients with advanced phase disease. Potential, yet controversial, mechanisms of resistance comprise *BCR-ABL* gene mutations, overexpression and amplification of the *BCR-ABL* gene locus [4], [5], activation of BCR-ABL independent pathways [6] or increased drug efflux [7], [8], [9], [10] as well as pharmacokinetic resistance [11]. Mutations in the kinase domain of *ABL,* which either prevent adoption of the inactive conformation required for IM binding or directly interfere with inhibitor interaction, have been identified as the predominant cause of relapse during IM therapy [12]. Second generation tyrosine kinase inhibitors, such as Dasatinib [13], Nilotinib [14] and Bosutinib [15], [16], are capable of overcoming the majority of resistance-conferring mutations clinically observed, with the exception of the highly IM-resistant gatekeeper mutation T315I. In addition, clinical observations as well as mathematical modeling approaches suggest that IM controls rather than cures CML [17], [18], [19], [20]. We have shown that immature CML cells are inherently insensitive to tyrosine kinase inhibitors, including IM [21], Dasatinib [22], Nilotinib [23] and Bosutinib [24]. Furthermore, persistence of minimal residual disease due to the limited effects of tyrosine kinase inhibitors on immature (quiescent) hematopoietic stem cells has been described [25]. Thus, the development of novel therapeutic strategies is a major goal in the treatment of CML, particularly in advanced stage and IM-resistant disease.

Recently, we reported on a novel small molecule inhibitor Danusertib (formerly PHA-739358), which exhibits potent efficacy against BCR-ABL and aurora kinases [26]. Anti-proliferative activity was observed in human leukemia cell lines as well as in CD34^+^ cells derived from newly diagnosed CML patients or IM-resistant individuals in chronic phase and blast crisis, including those harboring a T315I mutation [27], [28].

In this study, the individual contributions of BCR-ABL or aurora kinase inhibition to the anti-proliferative effect of Danusertib on Ph^+^ leukemic cells were analyzed. Particular interest was devoted to investigating the mechanisms of Danusertib resistance and the ability of combination therapy to prevent or reduce emergence of resistant clones *in vitro*.

## Results

### Inhibition of both BCR-ABL and aurora kinase pathways contributes to the effects of Danusertib

**Figure 1:**
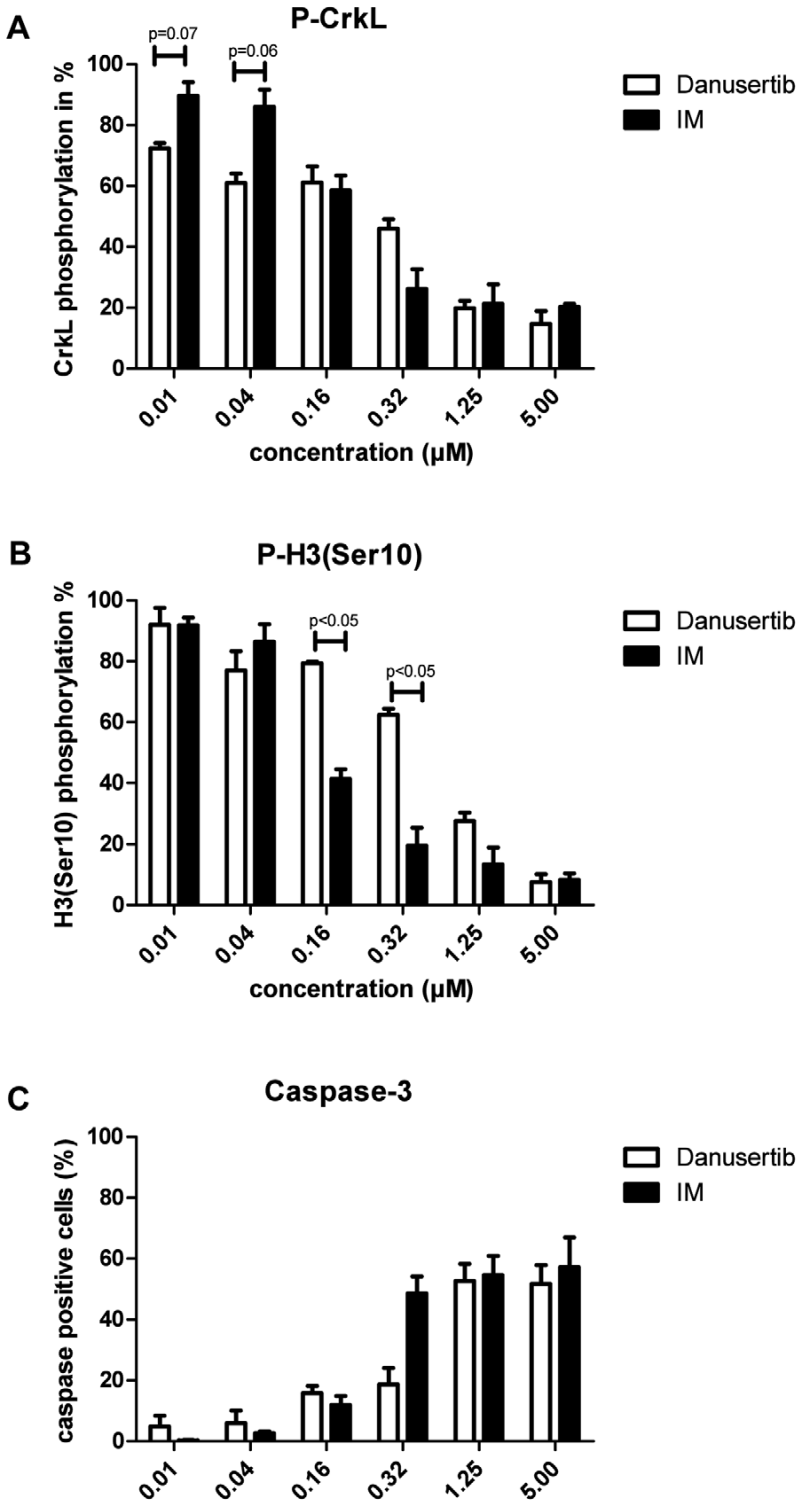
A–C: Contribution of BCR-ABL and aurora kinase inhibition to the pro-apoptotic effects of Danusertib. K562 cells were incubated with the indicated concentrations of Danusertib or IM for 24 hours. Intracellular flow cytometry was used to determine the phosphorylation status of well-known BCR-ABL and aurora kinase B downstream targets, CrkL (A) and histone H3-Ser10 (B), respectively. Induction of apoptosis was measured as the percentage of cells positive for activated caspase-3 (C). Bars represent the average of two independent experiments ± SD. Significance was determined using t-test.

We previously demonstrated that Danusertib inhibits both, aurora and ABL kinases, and induced anti-proliferative and pro-apoptotic effects via combined inhibition of the two respective pathways [27]. In order to further elucidate the particular contribution of the inhibition of either signal transduction pathway for the effect of Danusertib on *BCR-ABL*-positive cells, we treated K562 cells with Danusertib or IM at concentrations ranging from 0.01 to 5 µM for 24 hours and analyzed changes in the phosphorylation status of CrkL and histone H3-Ser10 (downstream target of BCR-ABL and aurora kinase B, respectively). As shown in Figure 1A, the use of Danusertib at the lowest, nanomolar concentration already resulted in some degree of inhibition of BCR-ABL activity. Gradually rising concentrations of Danusertib, up to 0.16 µM, were associated with a slowly increasing number of apoptotic cells. However, a marked reduction of CrkL phosphorylation, with a pronounced loss of viability due to apoptosis, was only observed for concentrations higher than 0.32 µM. Additionally, significant inhibition of phosphorylation of histone H3-Ser10 under Danusertib treatment was observed only at concentrations >0.32 µM when compared to P-CrkL (Figure 1B). However, for higher concentrations (>1.25 µM) strong inhibition of phosphorylation of CrkL and H3-Ser10 and a high degree of apoptosis were seen (Figure 1C). Taken togeter, these data suggest that the pro-apoptotic activity of Danusertib at lower concentrations is mainly due to inhibition of BCR-ABL kinase, whereas at higher concentrations inhibition of both BCR-ABL and aurora kinases pathways contributes to the observed effects. IM treatment at the lowest concentrations (0.01 and 0.04 µM) resulted in only slightly decreased phosphorylation of CrkL with no induction of apoptosis. However, starting from 0.16 µM strong inhibition of phosphorylation of CrkL with a more pronounced apoptosis was seen. A concomitantly decreased phosphorylation of H3-Ser10 observed under IM treatment may point to a predominant anti-proliferative effect preceding apoptosis as phosphorylation of H3-Ser10 by Aurora B is thought to be essential for chromatin condensation during cell division, and as such widely used as a marker of mitosis.

### Danusertib and IM synergize to induce apoptosis in *BCR-ABL*-positive IM-sensitive cell lines

**Figure 2:**
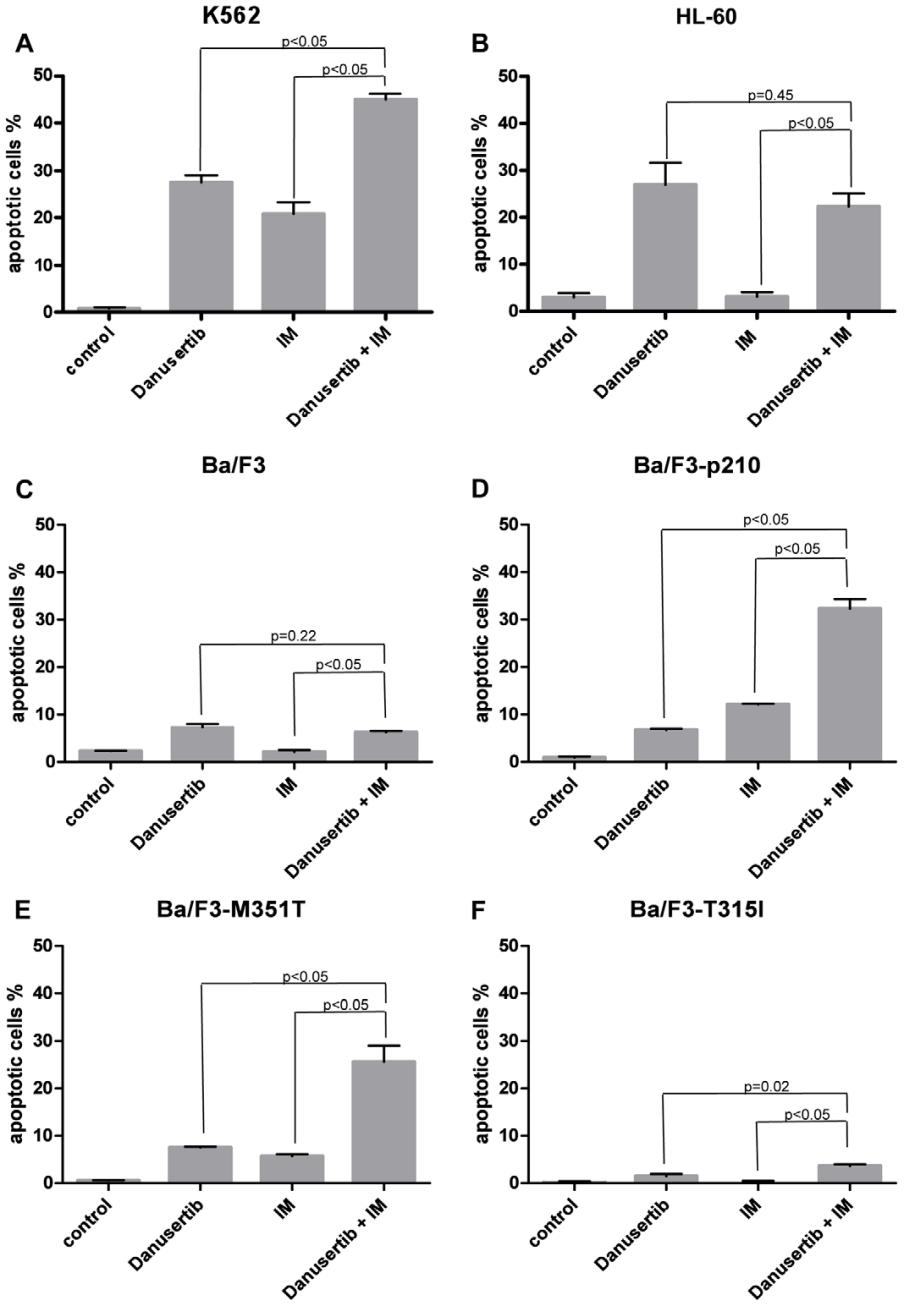
A–F: Danusertib and IM synergize to induce apoptosis in *BCR-ABL*-positive IM-sensitive cell lines. K562 (A), Ba/F3-p210 (D) and Ba/F3-M351T (E) cells were exposed to their respective IC_50_ concentrations of Danusertib (0.2, 0.4, and 0.5 µM, respectively), IM (0.2, 0.9, and 1.6 µM, respectively) or both inhibitors. HL-60 cells (B) were treated with their Danusertib IC_50_ concentration (3.0 µM), IM IC_50_ concentration for K562 or both whereas Ba/F3 (C) and Ba/F3-T315I (F) cells were exposed to their respective Danusertib IC_50_ concentrations (0.33 and 0.12 µM, respectively), IM IC_50_ concentration for Ba/F3-M351T (1.6 µM) or combination of both. After 24 hours cells were collected and the apoptotic fraction was assessed by flow cytometry as the percentage of cells positive for active caspase-3. Values for untreated controls were subtracted from the respective samples. Bar graphs represent mean % of positive cells of three experiments ± S.D. Significance was determined using t-test.

Simultaneous treatment with Danusertib and IM led to an increased rate of apoptosis in Ba/F3-p210, in the low-grade IM-resistant Ba/F3-M351T mutant, and in K562 cells, when compared to the single compounds. In contrast, no such effects were observed in *BCR-ABL*-negative Ba/F3, Ba/F3 harboring the highly IM-resistant T315I mutation, or in HL60 cells (Figure 2A–F).

### Danusertib induces polyploidy and cell death in stem and progenitor cells

Because of the known effects of Danusertib on aurora kinases [27], [29], additional cell cycle analysis was performed in CD34^+^ CML cells. A dose-dependent effect of Danusertib on the cell cycle profile was observed, resulting in an increase in polyploidy as well as a slightly rising number of apoptotic and necrotic cells, indicated by a growing subG1 peak (Figure S1). However, comparable to the findings of the apoptosis studies, these changes were most pronounced only at concentrations of Danusertib equal to or greater than 50 nM.

### Effects of Danusertib on different populations of hematopoietic stem and progenitor cells from CML patients

**Figure 3:**
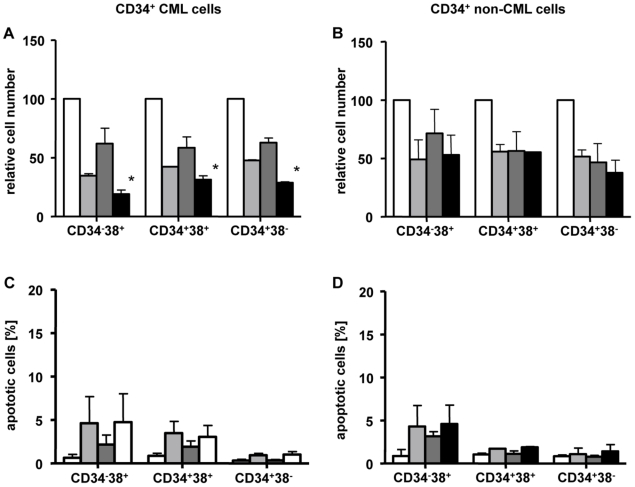
A–D: Danusertib is anti-proliferative against stem and progenitor cells independent of their differentiation or *BCR-ABL* status. CML and normal CD34^+^38^−^ primitive and CD34^+^38^+^ or CD34^−^38^+^ committed progenitors were exposed to IM (light grey), Danusertib (dark grey) or to the combination of both drugs (black) at indicated concentrations for 72 hours in relation to the control (white). Cell viability was assessed by trypan blue exclusion assay (A–B) and apoptosis was analyzed by FACS as the percentage of cells positive for active caspase-3 (C–D). The effects of Danusertib and IM on the viability of immature and committed CD34^+^ CML cells were found to be more pronounced for the drug combination (A). After treatment, small percentages (non significant) of active caspase-3 positive cells, in both *BCR-ABL*-positive and negative cells, were observed in the most committed fraction CD34^−^38^+^ (C–D). For the intermediate fraction, CD34^+^38^+^, a similar effect was seen for CML (C), but not for non-CML cells (D). No apoptosis was seen in the immature populations of either CML or non-CML cells. Results represent the mean ± S.D. of replicate experiments (n = 2) and asterices indicate p<0.05. Significance was determined using t-test.

**Figure 4:**
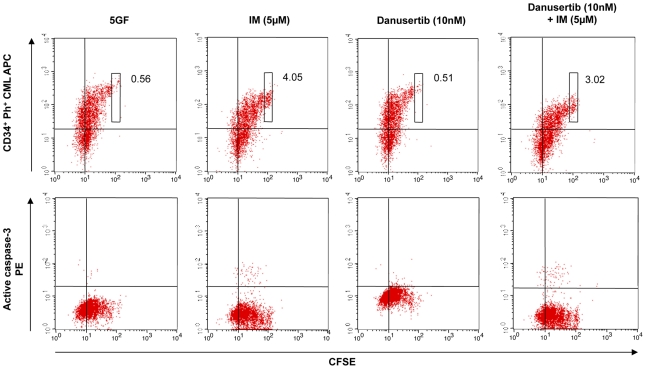
Cell division tracking using CFSE assay. Assessment of CD34 expression or active caspase-3 expression versus CFSE fluorescence remaining in cells following 72 hours of *in vitro* treatment with 5GF alone, 5 µM IM, 10 nM Danusertib and 5 µM IM+10 nM Danusertib. The boxes shown indicate undivided cells (CD34^+^, CFSE^max^) and the numbers represent the percent of total viable cells in this gate.

To further elucidate the efficacy of Danusertib on the different populations of hematopoietic stem and progenitor cells, hematopoietic cells derived from CML and non-CML patients were separated by a cell sorter into the following three subgroups: (1) CD34^+^38^−^ cells, (2) CD34^+^38^+^ cells, and (3) CD34^−^38^+^ cells, reflecting an increasing differentiation status of the cells. Following sorting, all three subgroups were treated with Danusertib, IM or a combination of the two compounds and the numbers of viable cells were evaluated by trypan blue exclusion assay. A significant reduction in the number of viable cells was demonstrated in all treatment groups following monotherapy with IM or Danusertib (Figure 3A–B). The combination of both drugs, however, significantly increased the observed effect on viable cells only for CML (Figure 3A). This effect was not limited to more differentiated cells, but was also detected in immature CD34^+^38^−^ cells (Figure 3A). As expected, no such additive effects were found in non-CML cells (Figure 3B) used as control. However, neither monotherapy with Danusertib (2 nM) nor IM (5 µM), nor the combination therapy caused significant induction of apoptosis in CD34^−^38^+^, CD34^+^38^+^ or CD34^+^38^−^ cells at the concentrations administered (Figure 3C–D). The effect of Danusertib on quiescent stem cells was then analysed in CFSE assays. CD34^+^ CML cells were treated with Danusertib (2 and 10 nM), IM (1 and 5 µM) or a combination of both compounds and viable cells were analysed. Again, the combination of both drugs induced a significant additive effect by reducing viable CD34^+^ cells. As described previously [30], IM resulted in an accumulation of CFSE^max^ cells, without significant induction of apoptosis, suggesting ineffective eradication of quiescent stem cells. At low concentrations (2 and 10 nM), Danusertib did not appear to significantly affect quiescent stem cells, either by accumulation of CFSE^max^ cells or by induction of apoptosis (Figure 4).

### Resistance to Danusertib is not mediated by mutations of the target kinases

**Figure 5:**
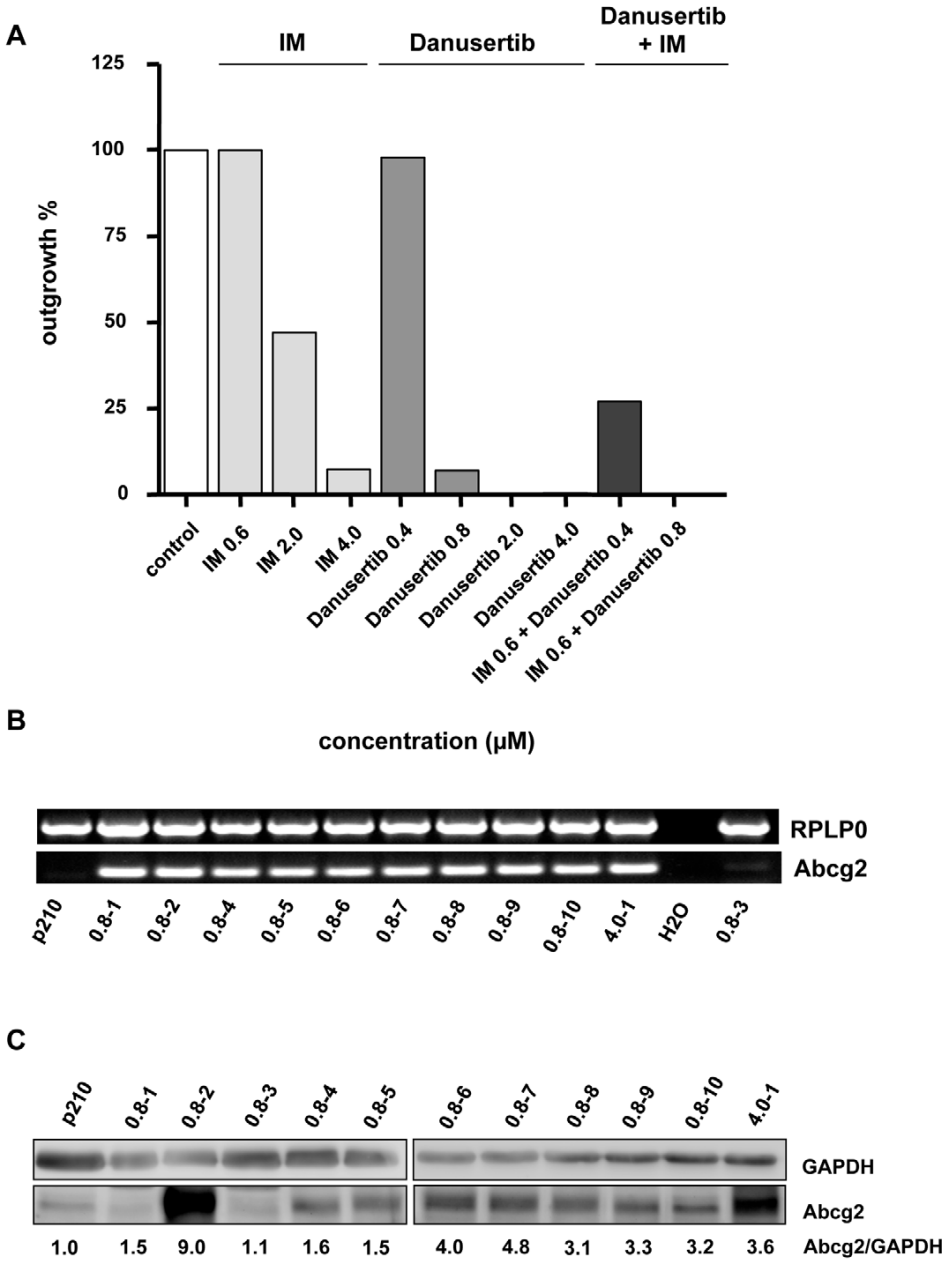
A–B: Danusertib induced Abcg2 overexpression in resistant clones and combination of Danusertib with IM reduces the frequency of resistant clones. (A) Ba/F3-p210 cells were cultured with graded concentrations of Danusertib or IM alone and in combination. Bars represent the percentage of wells from which drug-resistant clones were recovered. A total of 96 wells were analyzed in all experiments except those involving 0.8, 2, and 4 µM Danusertib as a single drug for which 144 wells were examined. (B) Abcg2 is overexpressed in all but one Danusertib-resistant clones. Semi-quantitative RT-PCR analysis for Abcg2 mRNA level in Danusertib-resistant clones and parental Ba/F3-p210 cells. (C) Abcg2 is overexpressed in all but two Danusertib-resistant clones. Western blotting analysis for Abcg2 level in Danusertib-resistant clones and parental Ba/F3-p210 cells. The relative expressions in comparison to GAPDH are indicated.

In order to generate a resistance profile for Danusertib, Ba/F3-p210 cells were treated with varying concentrations of Danusertib and IM, based on the assay published by von Bubnoff et al. [30]. In line with previous results, a dose-dependent development of resistance was observed for IM (Figure 5A). At the IC_50_ concentration 0.6 µM, 100% of the clones developed resistance in the course of long-term treatment (42d) *in vitro*. As expected, the frequency of resistant clones decreased with increasing concentrations of IM. Thus, exposure to 2 and 4 µM resulted in only 47% and 7.3% resistant clones, respectively. At a concentration of 2 µM, sequence analysis of the *ABL* kinase revealed only 3 of 96 clones that did not carry a mutation, while at higher concentration (e.g. 4 µM), mutations of the *ABL* kinase were detected in all 7 resistant clones. The mutational spectrum observed involved mutations that had previously been detected in patients receiving IM (e.g. Q252H, G250E, E316K, T315I), with the exception of the rather predominant A424T mutation (Figure S2).

A similar dose-dependent pattern of emergence of resistant clones was found for Danusertib. However, resistant clones occurred considerably less frequently compared to IM. Moreover, and in contrast to IM, under a concentration of 2 µM of Danusertib (i.e. 5×IC_50_) no resistant clones emerged and under 4 µM (i.e. 10×IC_50_,) literally one clone was detected over 42 days of treatment. Interestingly, sequence analysis of either *BCR-ABL* kinase or the coding sequences of aurora kinases A and B, did not reveal any mutations.

### Resistance to Danusertib is not associated with cross resistance to IM

To evaluate a possible cross resistance of Danusertib-resistant clones to IM, anti-proliferative activity of both compounds was analysed by MTT assay. As expected, a substantial increase of the IC_50_ values was observed for Danusertib (Table 1). However, IC_50_ of IM was not elevated suggesting a preserved sensitivity of the Danusertib-resistant cells for this tyrosine kinase inhibitor. In pharmacodynamic studies, BCR-ABL activity was assessed via the phosphorylation status of CrkL. For two clones showing a low-level of resistance for Danusertib (IC_50_: 2.1 and 2.8 µM, respectively) a reduced inhibition of P-CrkL was found when compared to Danusertib-sensitive cells. CrkL remained completely phosphorylated after treatment with Danusertib in highly resistant clones (IC_50_: 13.4 and 45 µM). In comparison, exposure to IM at a concentration of 5 µM led to the typical inhibition of P-CrkL in all clones analyzed suggesting effective inhibition of BCR-ABL kinase activity by this compound (Figure S3A–D).

**Table 1:** IC_50_ of Danusertib-resistant Ba/F3-p210 cells after long-term incubation with 0.8 µM (10 clones) or 4 µM (1 clone) of the inhibitor.

Clones and cell lines	IC_50_ Imatinib (µM)	IC_50_ Danusertib (µM)
0.8-1	0.9	2.1
0.8-2	0.6	13.4
0.8-3	0.8	2.8
0.8-4	0.3	1.1
0.8-5	0.3	2
0.8-6	0.5	1.2
0.8-7	0.7	1.2
0.8-8	0.6	1.2
0.8-9	1.2	5.3
0.8-10	0.8	9.5
4.0-1	1.6	45
Ba/F3-p210	0.6	0.4
Ba/F3	13.4	0.5

Interestingly, inhibition of the phosphorylation of histone H3-Ser10 was minimal in one of two low-level resistant clones compared to non-resistant cells and was virtually absent in other clones tested indicating an almost complete lack of Danusertib inhibitory effects on these serine/threonine kinases in resistant cells (data not shown).

### Combination therapy with Danusertib and IM considerably decreases the development of resistant clones in vitro

Next, the therapeutic impact of combination therapy with Danusertib and IM on *BCR-ABL*-positive cells was evaluated. Simultaneous use of both inhibitors considerably decreased the emergence of resistant clones in a dose-dependent manner. Thus, a combination of IM and Danusertib at their IC_50_ concentrations lowered the appearance of resistance to 27%, while in comparison, control cells treated with IC_50_ of either IM or Danusertib monotherapy, frequencies of 100 and 98% were observed, respectively. Remarkably, at the 2-fold IC_50_ concentration of Danusertib with IM at its IC_50_, a complete cell growth arrest was observed and no resistant clones were detected at the end of the experiment at day 42 (Figure 5A).

### Analysis of Microarray data reveals overexpression of Abcg2, a member of the ATP-binding cassette (ABC) superfamily, as a potential cause of Danusertib resistance

In order to screen for genes that might be involved in the emergence of Danusertib resistance, we hybridized samples from 2 resistant clones with the highest IC_50_ values and from parental cells onto Affymetrix GeneChip® Mouse Genome 430A 2.0 arrays. The statistical analysis of Microarray data allowed the identification of 138 and 181 differentially expressed genes in the first (0.8–2; IC_50_: 13.4 µM) and the second (4.0–1; IC_50_: 45 µM) resistant clone, respectively, as compared to the parental cells (Ba/F3-p210) with 95 genes common to both clones. Among them, Abcg2 gene was found to be about 1000-fold overexpressed in the resistant clones tested (Table 2). No other members of any transporter family were found significantly overexpressed and no other specific function was significantly affected. In view of these data, we examined the mRNA expression level of Abcg2 in all Danusertib-resistant clones. High Abcg2 overexpression was detected in all but one of the resistant clones analyzed by RT-PCR and in all but two of the resistant clones by Western blotting (Figure 5B–C). Thus, the observed Abcg2 up-regulation might account for the emergence of resistance under treatment with Danusertib.

**Table 2:** The top 10 up-regulated genes with the corresponding LogRatio values. In bold genes up-regulated in both analyzed clones.

Clone 0.8-2 Danusertib IC_50_: 13.4μ µM		Clone 4.0-1 Danusertib IC_50_: 45 µM	
Genes names	Exp. Value	Genes names	Exp. Value
**Abcg2**	**10.0**	**Abcg2**	**10.0**
GSTK1	2.7	**COL5A1**	**3.4**
**KEL**	**2.5**	CTTN	3.0
PIP	2.2	**KEL**	**2.3**
**TSC22D1**	**2.0**	**F10**	**2.2**
CD244	2.0	**TSC22D1**	**2.0**
**COL5A1**	**2.0**	MUC20	2.0
SERPINF1	2.0	CXCR4	1.9
**F10**	**1.9**	C10ORF54	1.8
ACSS1	1.6	ITPR2	1.8

### Pharmacological inhibition of Abcg2 altered the sensitivity of Danusertib-resistant clones

**Figure 6:**
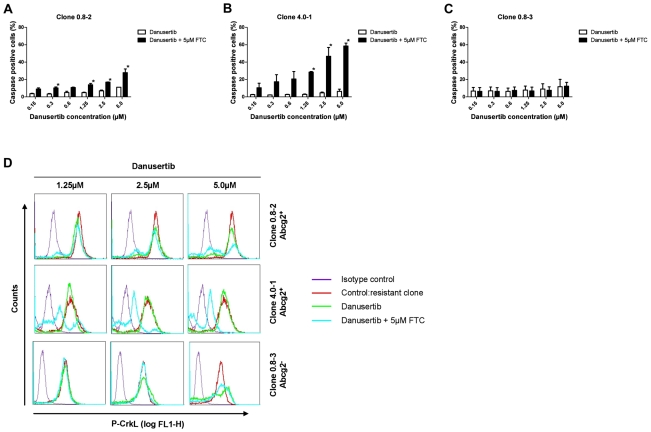
A–D: The Abcg2 inhibitor FTC overrides Danusertib resistance. Danusertib-resistant cells from two Abcg2-positive (A and B) and one Abcg2-negative (C) clones were exposed to indicated concentrations of Danusertib in the presence (black bar) or absence (white bar) of 5 µM FTC for 24 hours. The effects on induction of apoptosis were measured by active caspase-3 assay. Intracellular flow cytometric analysis was used to determine CrkL phosphorylation status (D). Results represent the mean ± S.D. of replicate experiments (n = 2) and asterices indicate p<0.05. Significance was determined using t-test.

Given the hypothesis that Abcg2 overexpression is responsible for resistance to Danusertib, we investigated whether the inhibition of Abcg2 activity with its specific inhibitor FTC was able to restore the effects of Danusertib treatment in vitro. Indeed, the cotreatment with FTC strongly enhanced the pro-apoptotic effects of Danusertib in resistant, Abcg2-overexpressing clones, whereas no such effects were seen in the single resistant Abcg2-negative clone (Figure 6A–C). Next, we evaluated the efficacy of Danusertib to inhibit BCR-ABL mediated phosphorylation of the downstream target CrkL in resistant clones cotreated with FTC. As shown in Figure 6D, Abcg2-overexpressing clones showed little or no changes in phosphorylation status of CrkL upon treatment with Danusertib alone whereas pronounced inhibition of CrkL-phosphorylation was observed in cells cotreated with FTC. As expected, no such effects were observed in Abcg2-negative control cells.

### Overexpression of Abcg2 in parental Ba/F3-p210 cells results in resistance to Danusertib

**Figure 7:**
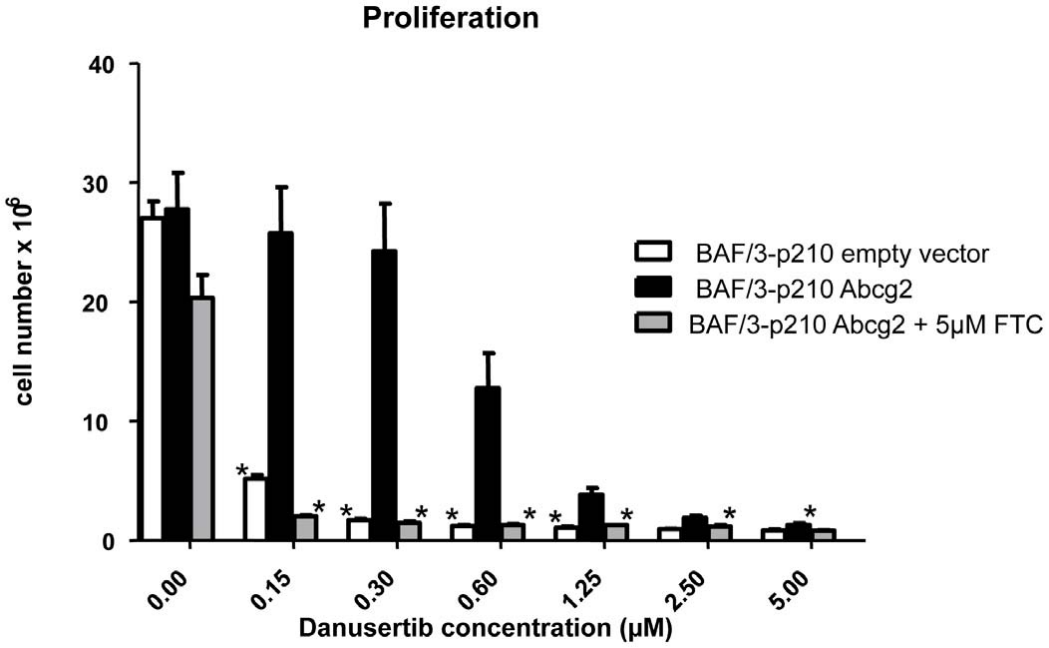
Retroviral overexpression of Abcg2 altered the sensitivity to Danusertib. Overexpression of Abcg2 in parental Ba/F3-p210 cells protects them against cytotoxic effects of Danusertib treatment. Ba/F3-p210 cells transduced with Abcg2 or with control empty vector were exposed for 48 hours to the indicated concentrations of Danusertib. In addition, the Abcg2-overexpressing Ba/F3-p210 cells were treated in the same way in the presence or absence of 5 µM FTC, a specific inhibitor of Abcg2 activity. In all experiments the number of viable cells was analyzed with Vi-CELL XR. Bar graphs represent the mean of three (empty vector and Abcg2) or six (Abcg2+5 µM FTC) experiments ± S.D. and asterices indicate p<0.05. Significance was determined using t-test.

In order to further validate that Danusertib is a substrate of the Abcg2 transporter, the effects of Danusertib treatment on cellular proliferation were evaluated in Ba/F3-p210 cells retrovirally transduced with Abcg2 or with control vector. The data from MTT assay clearly demonstrate that in cells overexpressing Abcg2, the IC_50_ for Danusertib was increased ∼4.5 fold as compared to control cells (IC_50_ values: 3.07 and 0.66 µM, respectively). In a short-term proliferation assay (48 hours) a strong dose-dependent reduction of cell growth was seen in control cells, whereas Abcg2-overexpressing cells were significantly less sensitive to Danusertib treatment (Figure 7). Again, this resistance-mediating effect of Abcg2 overexpression was almost completely abrogated when the cells were cotreated with FTC (Figure 7). Taken together, our data show that Danusertib is a substrate of the Abcg2 transporter and that overexpression of this efflux pump results in resistance to Danusertib which could be abrogated by the pharmacological inhibition of Abcg2 activity.

## Discussion

Danusertib, formerly PHA-739358, is a small molecule 3-aminopyrazole derivative which exhibits strong activity against all members of the aurora kinase family, as well as against ABL kinase [27], [29]. In crystallographic studies, Danusertib binds to an active conformation of the ABL kinase domain in the ATP-binding pocket [28]. Remarkably, Danusertib lacks the steric hindrance imposed by the substitution of threonine by isoleucine at position 315 and is therefore likely to overcome the highly IM resistance conferring T315I mutation. Indeed, we recently demonstrated that this inhibitor effectively targets wild type and mutant *BCR-ABL* positive cells, including the T315I mutation, *in vitro* [26] and *in vivo* [31]. Thus, Danusertib represents a promising new treatment option for patients suffering from IM-resistant CML and a phase II clinical trial, including CML patients in accelerated phase or blast crisis harboring the T315I mutation, is currently ongoing (NCT00335868, www.clinicaltrial.gov).

The aim of our study was to further characterize Danusertib with regard to its predominant mode of action (inhibition of BCR-ABL vs. aurora kinases) and its effects on the quiescent CD34^+^38^−^ hematopoietic stem cell subpopulation. In addition, particular focus was put on the potential development and underlying mechanism of resistance to Danusertib including the investigation of strategies suitable to overcome this problem.

First, in order to dissect the individual contribution of aurora and ABL kinase inhibition to the effect of Danusertib, changes in phosphorylation status of the BCR-ABL downstream target CrkL and the aurora kinase downstream target histone H3-Ser10 were analyzed. Exposure to Danusertib or IM at nanomolar concentrations resulted in inhibition of BCR-ABL activity. In comparison, inhibition of histone H3-Ser10 phosphorylation seemed to require higher amounts of Danusertib when compared to P-CrkL and distinct effects on the phosphorylation status were only observed at concentrations exceeding ∼0.16 µM. The inhibition of the BCR-ABL pathway seems to be the predominant effect of Danusertib in BCR-ABL positive cells *in vitro*, at least at low nanomolar concentrations.

These findings were further supported by cell cycle analysis. In line with the known effects of Danusertib on aurora kinases [27], [29], dose-dependent changes of the cell cycle profile were detected, reflected by increasing endoreduplication in both *BCR-ABL*-positive and negative CD34^+^ cells. However, these changes were only found at Danusertib concentrations exceeding 50 nM. Thus, we postulate that side effects due to aurora kinase inhibition in *BCR-ABL*-negative cells appear at higher dose levels, while in contrast, inhibition of proliferation and induction of apoptosis in *BCR-ABL*-positive cells can be achieved in the low nanomolar range, which might potentially result in an therapeutic index particularly for the treatment of highly IM-resistant BCR-ABL mutants such as T315I.

Preliminary findings from human and murine cell lines had pointed to a synergistic activity of Danusertib and IM [27]. Indeed, simultaneous treatment with Danusertib and IM resulted in pronounced apoptosis in K562, Ba/F3-p210, and Ba/F3 cells harboring the low-grade IM-resistant M351T mutation as compared to monotherapy. In contrast, no such effects were observed in *BCR-ABL*-negative cells or cells harboring the highly IM-resistant T315I mutation. In addition, the additive anti-proliferative activity of Danusertib and IM was also found in primary CD34^+^ cells derived from CML patients, including the immature CD34^+^38^−^ subpopulation which has been demonstrated to contain quiescent leukemic stem cells [32], [33].

In order to achieve cure of CML, the effective eradication of minimal residual disease (e.g. quiescent CML stem cells) remains an urgent treatment goal. However, neither IM nor second generation tyrosine kinase inhibitors such as Dasatinib, Nilotinib or Bosutinib has so far been demonstrated to effectively induce apoptosis in the quiescent stem cell compartment [34], [35]. Insufficient drug concentrations due to an imbalance between drug influx and efflux [34], [35], gene amplification [4], increase of activity of BCR-ABL downstream effector kinases or high *BCR-ABL* transcripts have been discussed as possible mechanisms [36], [37]. Indeed, the number of *BCR-ABL* copies and transcript levels were found to be significantly higher within CD34^+^38^−^ CML cells suggesting an increased activity of the BCR-ABL protein in this cell subpopulation [22]. In line with previous findings, we did not observe a loss of cell viability in stem or progenitor cells treated with IM. Furthermore, and in contrast to the proliferation experiments, neither Danusertib alone nor in combination with IM was capable of inducing significant apoptosis in the stem cell compartment when administered at low nanomolar concentrations. In line with this finding, Danusertib did not affect quiescent stem cells in CFSE assays *in vitro*. These results point to a pronounced anti-proliferative effect, but do not support significant activity towards eradication of quiescent and/or minimal residual disease.

Occurrence of resistance secondary to treatment with tyrosine kinase inhibitors complicates treatment of CML and is the major cause of disease relapse in the course of therapy. In order to generate a possible resistance profile, Ba/F3-p210 cells were treated with varying concentrations of Danusertib or IM. Confirming previous results, a dose-dependent development of resistance was observed for IM with a mutational spectrum similar to mutations observed clinically with the exception of the rather predominat A424T mutation which, to our knowledge, has not been described so far. Similarly, treatment with Danusertib caused resistance in a dose-dependent manner however, resistant clones occurred considerably less frequently than with IM treatment. In support of these data, clones showing low-level resistance to Danusertib exhibited a strong inhibition of phosphorylation of the downstream target CrkL but interestingly, only minimal inhibition of histone H3-Ser10 phosphorylation under Danusertib treatment. In highly resistant clones, CrkL and histone H3-Ser10 remained completely phosphorylated, indicating loss of efficacy of Danusertib against both BCR-ABL and aurora kinases. Remarkably, all Danusertib-resistant cells remained IM-sensitive as indicated by MTT assays as well as by preservation of inhibition of CrkL-phosphorylation under IM. Furthermore, sequence analysis of *BCR-ABL* kinase domain and the coding sequences of aurora kinases A and B, revealed no mutations and therefore was eliminated as a possible mechanism of Danusertib resistance. These findings strongly pointed to a common mechanism underlying resistance to Danusertib in Ba/F3-p210 cells independent of the individual target kinases. Analysis of data from microarray gene expression profiling of resistant clones and biochemical functional assays revealed that overexpression of the Abcg2 efflux transporter protein was responsible for the emergence of resistance under Danusertib treatment in 10 out of 11 clones. The functional relevance of these findings was strongly supported by studies using the pharmacological Abcg2-inhibitor FTC. Indeed, clones resistant to Danusertib and overexpressing Abcg2 became sensitive to drug again once cotreated with FTC whereas this was not the case in the single resistant clone which did not overexpress Abcg2. Furthermore, retroviral overexpression of Abcg2 in BCR-ABL-expressing Ba/F3 cells lead to resistance of the cells to Danusertib which in turn, could again be reversed by cotreatment with FTC. Taken together theses findings suggest a central role of drug efflux out of BCR-ABL^+^ cells mediated by Abcg2 overexpression as the mechanism of resistance to Danusertib.

In view of the diverse and non-overlapping mechanisms of resistance found for Danusertib as opposed to IM, the impact of a combination therapy of both compounds was addressed in long-term expansion culture. Simultaneous administration of both inhibitors considerably decreased occurrence of resistance in a dose-dependent manner. Noteworthy, at the twofold IC_50_ concentration of Danusertib combined with IM at IC_50_, development of resistance could be prevented altogether.

In conclusion, Danusertib potently inhibits proliferation of *BCR-ABL*-positive CD34^+^ stem and progenitor cells including the immature CD34^+^38^−^ subpopulation. However, similarly to other tyrosine kinase inhibitors, no induction of apoptosis in quiescent hematopoietic stem cells could be achieved under Danusertib *in vitro*. Resistant *BCR-ABL*-positive clones emerged in the course of Danusertib treatment and the predominant mechanism of Danusertib resistance was overexpression of Abcg2, which could be effectively overcome by pharmacological inhibition of the activity of this efflux transporter. Furthermore, Danusertib-resistant clones retained full sensitivity to IM and the combination of Danusertib and IM successfully prevented emergence of resistant clones altogether. These results suggest that Danusertib is a substrate for the Abcg2 drug transporter. With regards to the known inhibitory effects of IM on Abcg2, our studies suggest that the additive effects of Danusertib and IM on the emergence of resistant clones *in vitro* and on proliferation of primary CML stem and progenitor cells can be based on the accumulation of Danusertib in Abcg2 expressing cells [10]. Recent in vitro studies from Hiwase et al. have identified a similar mechanism for the combination of Nilotinib and Dasatinib in CML cells overexpressing the Abcb1 efflux protein [38].

Thus, these results represent a strong rationale for follow-up studies addressing combination therapies of Danusertib with IM and potentially, 2nd generation tyrosine kinase inhibitors such as Nilotinib, Dasatinib and Bosutinib in order to improve therapeutic effects in *BCR-ABL*-positive leukemias.

## Materials and Methods

### Reagents

IM was purchased from Toronto Research Chemicals Inc, Ontario, Canada. Danusertib (formerly PHA-739358) was obtained from Nerviano (Milan, Italy). Stock solutions of IM (10 mg/mL; in DMSO/H_2_O (1∶1)) and Danusertib (10 µM; in DMSO) were stored at −20°C. Fumitremorgin C (FTC) was obtained from Alexis Biochemicals, San Diego, CA, USA and prepared as a 5 mM stock solution in DMSO.

### Cell culture techniques

Human cell lines K562, HL60, and murine Ba/F3 were obtained from DSMZ (Bielefeld, Germany). Ba/F3-p210, -M351T, and -T315I cells were obtained from N.P. Shah and C.L. Sawyers (UCLA, USA). All cell lines were cultured under standard conditions as previously described [27].

### Isolation of CD34^+^ cells and their subpopulations

Fresh leukapheresis or peripheral blood samples were obtained with written informed consent and approval of North Glasgow University Hospital Division of NHS Greater Glasgow Institution Review Board from patients with newly diagnosed chronic phase CML (n = 4) and normal donors of peripheral blood stem cells (n = 2). Samples were enriched for CD34^+^ cells using CliniMACS (Miltenyi Biotec, Auburn, CA). CD34^+^ cells were expanded *in vitro* as described previously [22].

### Mutagenesis screen

Ba/F3-p210 cells were cultured in 24-well plates at a density of 1×10^5^ cells per well in the presence of 0.6, 2 and 4 µM IM or 0.4, 0.8, 2 and 4 µM Danusertib. For the combination assays, IM was used at a fixed concentration of 0.6 µM with 0.4 or 0.8 µM Danusertib. Single colonies growing out on the bottom of wells were expanded for further analysis in the presence of inhibitor at the concentration corresponding to that used in the screen. To identify possible mutations in aurora kinase A and B domains, the coding cDNAs of both enzymes were sequenced using the following primers: auroraA1-fwd 5′-AGGTTCCTGCCTGTGAGTTG-3′; auroraA1-rev 5′-GCTCATTCTTCTGGGGGTTA-3′; auroraA2-fwd 5′-GCAGATTCCGTCTCAGAACC-3′; auroraA2-rev 5′-GCTGTTCTCTGCTCGTCAAA-3′; auroraA3-fwd 5′-GAGAGGTGGAGATCCAGTCG -3′; auroraA3-rev 5′-TGCATGGCCAAGGACTAGTT-3′; auroraB1-fwd 5′-CCTCTGCATCTCTTGCCTTC-3′; auroraB1-rev 5′-CTCCCTGCAGACCTAACAGC-3′, auroraB2-fwd 5′-CCTGAAACATCCCAACATCC-3′, and auroraB2-rev 5′- TTCCCACCCCTTCTCAGAAC-3′. Sequencing of the ABL kinase domain was performed as previously described [31]. For all RT-PCR reactions, total RNA was isolated using TRIzol (Invitrogen). cDNA was prepared by reverse transcription of 250 ng RNA with oligo(dT)_15_ and Superscript II reverse transcriptase (Invitrogen) and amplified using REDTaq™ ReadyMix™ PCR Reaction Mix (Sigma-Aldrich).

### RNA isolation and measurement of Abcg2 transcripts by semiquantitative RT-PCR

RNA was isolated from the different cell clones using TRIzol® reagent (Invitrogen, Karlsruhe, Germany) according to the manufacturers' protocol. The cDNA was prepared by reverse transcription of 1 µg total RNA using oligo dT primer (15mer) and M-MLV reverse transcriptase (Fermentas Life Sciences GmbH, Germany). The PCR for Abcg2 and for the RPLP0 housekeeping genes were performed as follow: 1 cycle of 94°C for 4 min and 30 cycles of 94°C for 30 s, 58°C for 1 min and 72°C for 1 min. For amplification following primer pairs were used: for Abcg2 - mAbcg2-fw: 5′ -TCG CAG AAG GAG ATG TGT TG- 3′ and mAbcg2-rev: 5′ –TTG GAT CTT TCC TTG CTG CT- 3′ which gives a 204-bp product and for RPLP0 - RPLP0-fw: 5′ -TTG TGT TCA CCA AGG AGG AC- 3′ and RPLP0-rev: 5′ -GAC TCT TCC TTG GCT TCA AC- 3′ which gives a 649-bp product.

### Western Blotimg

Protein extracts were separated by 12% SDS-PAGE and electrophoretically transferred onto PVDF membrane. Blocking was carried out in 1×Rotiblock solution (Carl Roth, Karlsruhe, Germany) followed by incubating the membrane with anti-Abcg2 antibody (Santa Cruz Biotechnology, Santa Cruz, CA) and anti-GAPDH antibody (Millipore, Billerica, MA) overnight at 4°C. Excess antibodies were removed by washing with NaCl–Tris–Tween 20. Incubation with secondary antibody conjugated to horseradish peroxidase [anti-(mouse IgG) or anti-(rabbit IgG), diluted 1∶5000 in 1×Rotiblock] was performed for 1 h at room temperature. After three washes, the reaction was developed by the addition of SuperSignal West Pico Chemiluminescent Substrate (Thermo Fisher Scientific, Rockford, IL). The emitted light was captured on X-ray film (GE Healthcare, Uppsala, Sweden). Relative expression of Abcg2 was estimated using Quantity One software (Bio-Rad Laboratories, Hercules, CA).

### Short-term proliferation and MTT assay

For a short-term (48 hours) proliferation assay, cells were collected and analyzed by Vi-CELL XR (Beckman Coulter, Krefeld, Germany) according to the manufacturer's recommendation. The 3-(4,5-dimethylthiazol-2-yl)-2,5-diphenyltetrazolium bromide (MTT) assay was performed as previously described [39].

### Intracellular flow cytometry

Assessment of protein expression level or phosphorylation status of Crkl and histone H3-Ser10 by intracellular flow cytometry was performed as described elsewhere [27].

### Analysis of apoptosis by flow cytometry

K562, Ba/F3-p210 and Ba/F3-M351T cells were plated in triplicates and exposed to their IC_50_ concentrations for Danusertib (0.2, 0.4, and 0.5 µM, respectively), IM (0.2, 0.9, and 1.6 µM, respectively) or both inhibitors, whereas HL-60, Ba/F3, and Ba/F3-T315I cells were treated with their respective Danusertib IC_50_ concentrations (3.0, 0.33, and 0.12 µM, respectively), IM IC_50_ concentration for K562 (HL-60) and for Ba/F3-M351T (Ba/F3 and Ba/F3-T315I) or combination of both. Following 24-hour treatment, cells were fixed and permeabilized (Ba/F3 cells with paraformaldehyde and methanol; other cells with FIX & PERM®, Invitrogen), stained with an anti-active caspase-3 PE-conjugated antibody (BD Biosciences) and analyzed by flow cytometry.

### Analysis of DNA content by flow cytometry

Flow cytometric analysis of DNA content using propidium iodide was performed as previously described [34], [35].

### Tracking of cell division using carboxyfluorescein diacetate succinimidyl diester (CFSE) staining

CD34^+^ cells were stained with 1 µM CFSE (Invitrogen) as described previously [22], [40] and cultured in the presence of different drug combinations. Total cell viability was assessed using trypan blue exclusion. Cells cultured with 100 ng/mL colcemid (Invitrogen) were used to establish the CFSE^max^ quiescent cell population at all time points. CFSE^+^ cells of each condition were stained with an anti-CD34-APC-conjugated antibody and anti-active caspase-3-PE-conjugated antibody (BD PharMingen, San Diego) for quantification of apoptosis. The number of quiescent cells was determined by measuring the percentage recovery of the starting number of viable CD34^+^ cells in the CFSE^max^ quiescent subpopulation as described previously [21].

### Sample preparation and microarray gene expression profiling

Microarray gene expression profiling experiments were performed on cell culture triplicates for the parental Ba/F3-p210 cell line and for two independent Danusertib-resistant clones, respectively. Total RNA was extracted from frozen cell pellets (8–10×10^6^ cells for each cell culture replicate) using the RNeasy Mini Kit (Qiagen, Hilden, Germany), after homogenization with QIAshredder columns (Qiagen), according to the manufacturer's instructions. Biotin-labeled, fragmented cRNA probes were prepared starting from 1.5 µg of total RNA per replicate sample, using the ‘One-Cycle Target Labeling and Control Reagents’ (Affymetrix, Santa Clara, CA) according to the protocols by Affymetrix GeneChip Expression Analysis Technical Manual. Samples were hybridized onto Affymetrix GeneChip® Mouse Genome 430A 2.0 arrays (representing approximately 14000 well-characterized mouse genes) and processed as per manufacturer's instructions using ‘GeneChip® Hybridization, Wash, and Stain Kit’ components (Affymetrix; www.affymetrix.com). Microarray data analysis was performed using Bioconductor libraries (www.bioconductor.org). Probe set intensity were calculated using the RMA algorithm and normalized by the quantile method [41], [42]. LIMMA statistical analysis was performed in order to identify differentially expressed probe sets (pV<0.05, FC>2). Annotation and functional analysis were performed using Ingenuity Pathways Analysis (IPA) 7.1 software (Ingenuity® System; www.ingenuity.com), a commercial database containing manually annotated data for human protein-protein and functional interactions derived from the literature.

### Pharmacological inhibition of Abcg2-mediated resistance to Danusertib

Cells from two Abcg2-positive and one Abcg2-negative Danusertib-resistant clones were plated in 24-well plates (0.3×10^6^/well) and treated for 24 hours with different concentrations of Danusertib (0.15–5 µM) in the absence or presence of 5 µM FTC. After fixation and permeabilization, cells were tested for apoptosis induction and changes in CrkL-phosphorylation status.

### Cloning of murine Abcg2 and retroviral transduction procedure

Murine Abcg2 was PCR-cloned from the mouse Abcg2 cDNA clone (Origene, Rockville, USA) into the MSCV-puro-vector system (Clontech, Moutain View, USA). Retroviral supernatant was generated using ecotropic Phoenix cells (ATCC, Manassas, USA) cultured in Dulbecco's Modified Eagle's Medium (DMEM, Invitrogen) supplemented with 10% FBS. Ba/F3 and Ba/F3-p210 cells were transduced with Abcg2-puro or the corresponding empty-vector using a calcium phosphate transfection protocol. Briefly, 20 µg DNA were mixed with 62.5 µl 2 M CaCl_2_ and adjusted to 500 µL with sterile H_2_O. 500 µL Hepes-buffered saline (2× HBS) was slowly added to the DNA solution under constant air agitation. Afterwards, the solution was mixed with medium, including 25 µM chloroquine and added to ∼6×10^6^ Phoenix cells. After incubation over night, medium was replenished to collect virus supernatant after 24, 36 and 48 hours and supplemented with 4 µg/mL polybrene. After transduction, cells were selected with puromycin at a final concentration of 2.5 µg/mL.

## Supporting Information

Figure S1 **Danusertib induces apoptosis, accumulation of cells with more than or equal to 4N DNA content and G2/M arrest in** ***BCR-ABL*** **-positive CD34^+^ cells.** CD34^+^ cells were exposed for 72 hours to indicated concentrations of Danusertib with (lower panels) or without (upper panels) 1 µM IM. Analysis of cell cycle and apoptotic/necrotic fraction of propidium iodide-stained cells was assessed by flow cytometry.
(TIF)

Figure S2 **The frequency and mutational spectrum observed in IM-resistant clones.** Exposure to 2 µM (i.e. 5×IC_50_) and 4 µM (i.e. 10×IC_50_) resulted in 47 and 7.3% resistant clones, respectively. Mutations found in the ABL kinase domain had already been detected in IM-treated patients, with the exception of the A424T mutation.
(TIF)

Figure S3 **Danusertib resistant cells do not show cross resistance to IM.** Cells from different Danusertib-resistant clones, cultivated under resistance generating conditions, were exposed to 5 µM Danusertib or IM for 24 hours. Intracellular flow cytometric analysis was used to determine CrkL phosphorylation status.
(TIF)
