# Supplementary figures and images for: Abcg2 Overexpression Represents a Novel Mechanism for Acquired Resistance to the Multi-Kinase Inhibitor Danusertib in BCR-ABL-Positive Cells In Vitro
Source: PLoS One. 2011 Apr 26;6(4):e19164. doi: 10.1371/journal.pone.0019164 (PMC3082549; doi:10.1371/journal.pone.0019164)

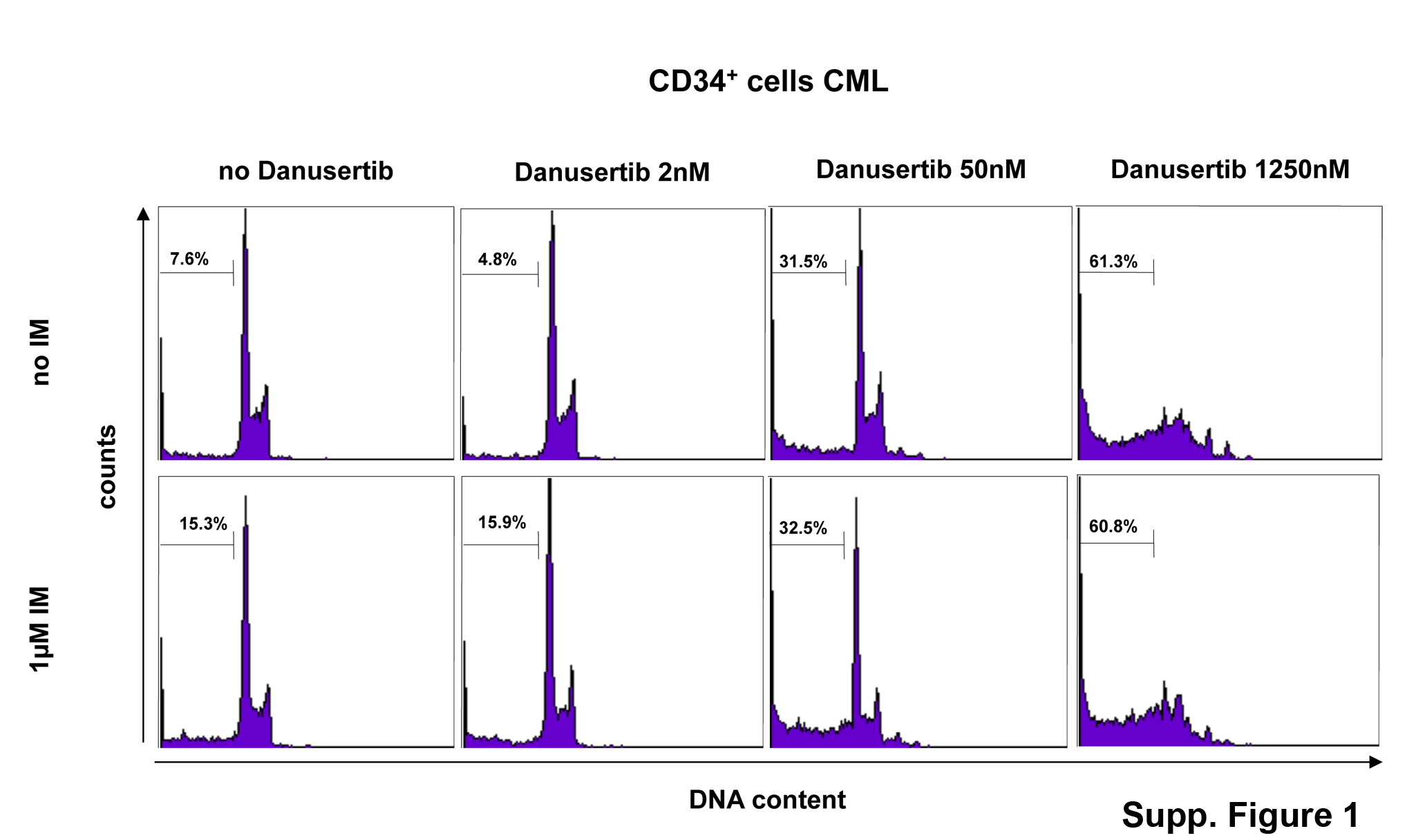

Supplement: Figure S1 — Danusertib induces apoptosis, accumulation of cells with more than or equal to 4N DNA content and G2/M arrest in BCR-ABL -positive CD34+ cells. CD34+ cells were exposed for 72 hours to indicated concentrations of Danusertib with (lower panels) or without (upper panels) 1 µM IM. Analysis of cell cycle and apoptotic/necrotic fraction of propidium iodide-stained cells was assessed by flow cytometry. (TIF) [file pone.0019164.s001.tif]

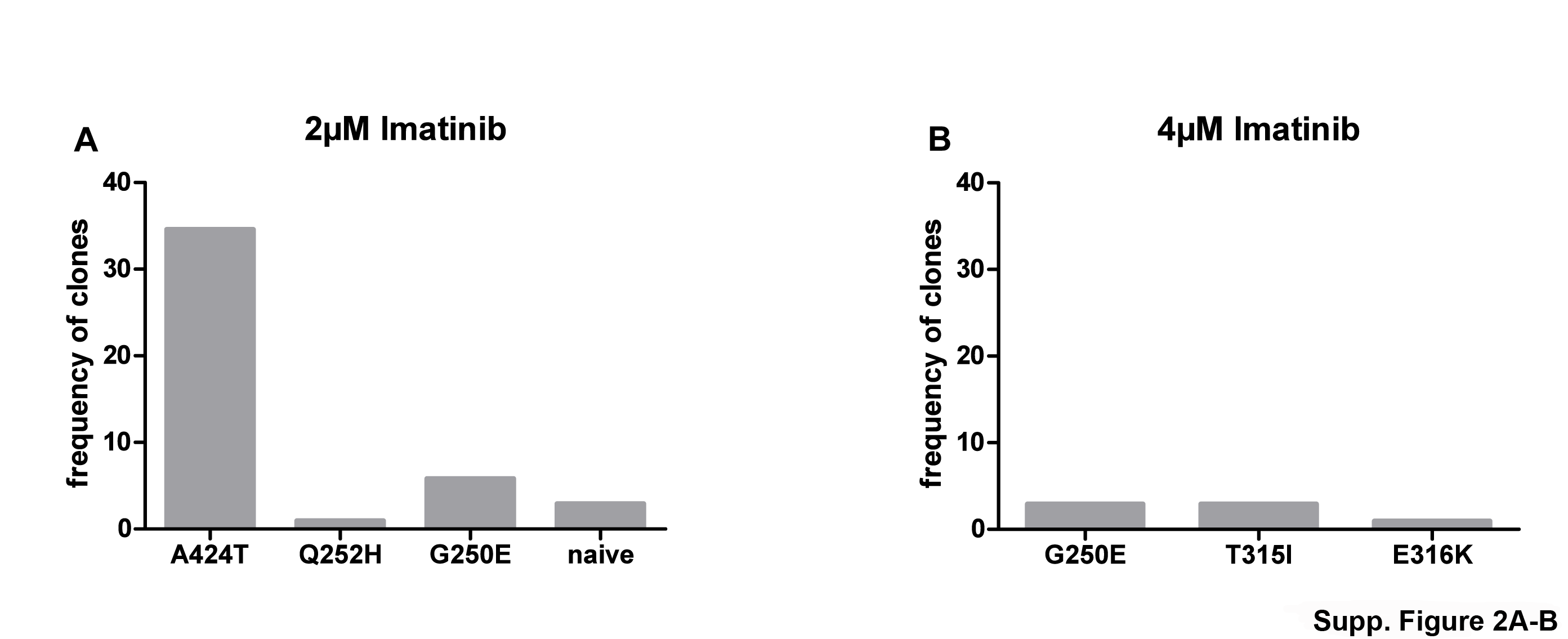

Supplement: Figure S2 — The frequency and mutational spectrum observed in IM-resistant clones. Exposure to 2 µM (i.e. 5×IC50) and 4 µM (i.e. 10×IC50) resulted in 47 and 7.3% resistant clones, respectively. Mutations found in the ABL kinase domain had already been detected in IM-treated patients, with the exception of the A424T mutation. (TIF) [file pone.0019164.s002.tif]

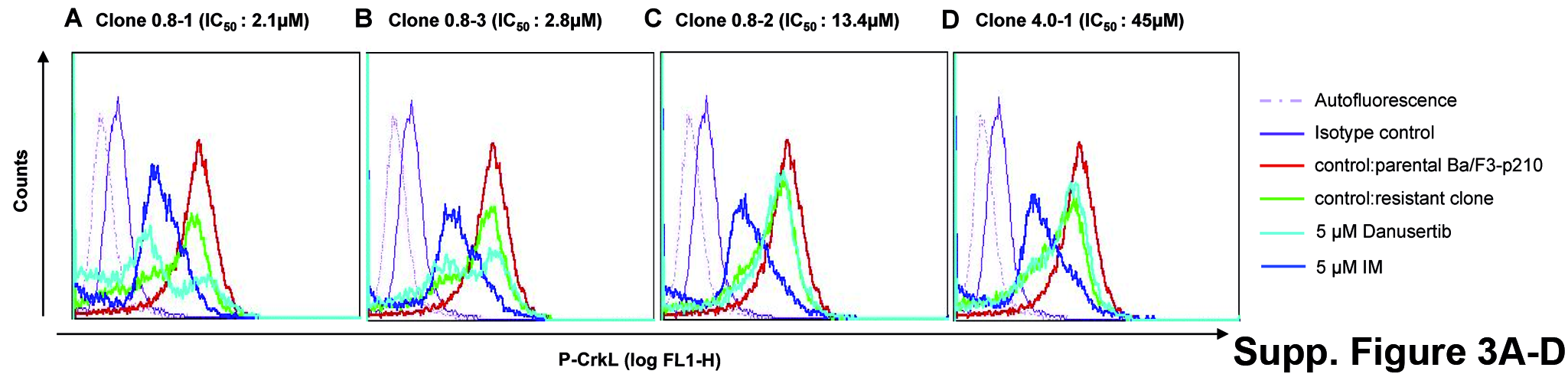

Supplement: Figure S3 — Danusertib resistant cells do not show cross resistance to IM. Cells from different Danusertib-resistant clones, cultivated under resistance generating conditions, were exposed to 5 µM Danusertib or IM for 24 hours. Intracellular flow cytometric analysis was used to determine CrkL phosphorylation status. (TIF) [file pone.0019164.s003.tif]
